# Fluoxetine Does Not Enhance Visual Perceptual Learning and Triazolam Specifically Impairs Learning Transfer

**DOI:** 10.3389/fnhum.2016.00532

**Published:** 2016-10-19

**Authors:** Alice K. Lagas, Joanna M. Black, Winston D. Byblow, Melanie K. Fleming, Lucy K. Goodman, Robert R. Kydd, Bruce R. Russell, Cathy M. Stinear, Benjamin Thompson

**Affiliations:** ^1^School of Optometry and Vision Science, University of AucklandAuckland, New Zealand; ^2^Centre for Brain Research, University of AucklandAuckland, New Zealand; ^3^Department of Exercise Sciences, University of AucklandAuckland, New Zealand; ^4^Centre of Human and Aerospace Physiological Sciences, King’s College LondonLondon, UK; ^5^Department of Psychological Medicine, University of AucklandAuckland, New Zealand; ^6^School of Pharmacy, University of AucklandAuckland, New Zealand; ^7^National School of Pharmacy, University of OtagoDunedin, New Zealand; ^8^Department of Medicine, University of AucklandAuckland, New Zealand; ^9^School of Optometry and Vision Science, University of Waterloo, WaterlooON, Canada

**Keywords:** motion perception, serotonin uptake inhibitors, learning, TMS, mood, phosphene threshold, motor threshold, paired associative stimulation (PAS)

## Abstract

The selective serotonin reuptake inhibitor fluoxetine significantly enhances adult visual cortex plasticity within the rat. This effect is related to decreased gamma-aminobutyric acid (GABA) mediated inhibition and identifies fluoxetine as a potential agent for enhancing plasticity in the adult human brain. We tested the hypothesis that fluoxetine would enhance visual perceptual learning of a motion direction discrimination (MDD) task in humans. We also investigated (1) the effect of fluoxetine on visual and motor cortex excitability and (2) the impact of increased GABA mediated inhibition following a single dose of triazolam on post-training MDD task performance. Within a double blind, placebo controlled design, 20 healthy adult participants completed a 19-day course of fluoxetine (*n* = 10, 20 mg per day) or placebo (*n* = 10). Participants were trained on the MDD task over the final 5 days of fluoxetine administration. Accuracy for the trained MDD stimulus and an untrained MDD stimulus configuration was assessed before and after training, after triazolam and 1 week after triazolam. Motor and visual cortex excitability were measured using transcranial magnetic stimulation. Fluoxetine did not enhance the magnitude or rate of perceptual learning and full transfer of learning to the untrained stimulus was observed for both groups. After training was complete, trazolam had no effect on trained task performance but significantly impaired untrained task performance. No consistent effects of fluoxetine on cortical excitability were observed. The results do not support the hypothesis that fluoxetine can enhance learning in humans. However, the specific effect of triazolam on MDD task performance for the untrained stimulus suggests that learning and learning transfer rely on dissociable neural mechanisms.

## Introduction

Visual perceptual learning (VPL) is an established model of adult human brain plasticity that involves improved visual task performance with training (Epstein, 1967; Gibson, 1969, 1991; Watanabe and Sasaki, 2015). VPL can be highly specific to the trained stimulus configuration (Ball and Sekuler, 1982). This implies the involvement of neural populations in visual areas such as V1, V2, V4, and the middle temporal area (MT) that are tightly tuned to orientation, retinal location and motion direction (Fiorentini and Berardi, 1980; Ball and Sekuler, 1982, 1987; Karni and Sagi, 1991; Gilbert, 1994). The involvement of the primary and extrastriate visual cortex in VPL has been supported, in part, by primate neurophysiology (Zohary et al., 1994; Crist et al., 2001; Schoups et al., 2001; Ghose et al., 2002; Li et al., 2004, 2008; Rainer et al., 2004; Yang and Maunsell, 2004; Raiguel et al., 2006; Hua et al., 2010; Adab and Vogels, 2011; Yan et al., 2014) and human neuroimaging studies (Schiltz et al., 1999, 2001; Schwartz et al., 2002; Furmanski et al., 2004; Walker et al., 2005; Yotsumoto et al., 2008; Bao et al., 2010; Shibata et al., 2011, 2012; Thompson et al., 2013; Bi et al., 2014). However, transfer of learning to untrained stimuli and tasks can occur (Ahissar and Hochstein, 1997; Liu, 1999; Liu and Weinshall, 2000; Xiao et al., 2008; Jeter et al., 2009; Green et al., 2015) indicating that higher-level decision making areas are also involved in VPL. In agreement with this idea, primate neurophysiological data have revealed learning induced changes within the decision-making area LIP (lateral intraparietal sulcus) but not in MT following motion discrimination VPL (Law and Gold, 2008). Neuromodulation and fMRI studies in humans have also shown that VPL involves decision-making areas (Kahnt et al., 2011; Chen et al., 2015, 2016).

In accordance with evidence suggesting that VPL involves multiple cortical areas, some current VPL models propose changes at more than one stage of visual processing or type of stimulus representation (Dosher et al., 2013; Watanabe and Sasaki, 2015). For example, Watanabe and Sasaki (2015) suggest that perceptual learning of a trained task involves changes in the representation of stimulus features, possibly within visual cortex, as well as changes in task-specific visual processing that may involve decision making areas. Within this schematic model, transfer of learning to an untrained stimulus location may involve task-specific changes only (Watanabe and Sasaki, 2015).

Visual perceptual learning has been used as a model to investigate the effects of neuromodulatory interventions on adult brain plasticity. Interventions that enhance VPL include transcranial random noise stimulation (Fertonani et al., 2011) and the administration of donepezil to increase the synaptic concentration of acetylcholine (Rokem and Silver, 2010, 2013). Enhanced VPL was also found in patients with increased glutamatergic transmission due to Huntington’s disease (Beste et al., 2012). These studies demonstrate that altered cortical function can enhance VPL.

Pharmacological manipulations have also been used to increase visual cortex plasticity in animal models (Bavelier et al., 2010). In an influential study, Maya Vetencourt et al. (2008) found that chronic administration of the selective serotonin reuptake inhibitor (SSRI) fluoxetine dramatically enhanced visual cortex plasticity in adult rats, effectively ‘reopening’ the critical period for visual cortex development. Plasticity was linked to an increased concentration of brain derived neurotrophic factor (BDNF) and a reduction in visual cortex gamma-aminobutyric acid (GABA) concentration. Increasing GABA with diazepam blocked the plasticity enhancing effects of fluoxetine. This result suggested that fluoxetine might be beneficial in the treatment of neurological vision disorders such as amblyopia in adult patients.

Selective serotonin reuptake inhibitors may also increase cortical plasticity in humans. Normann et al. (2007) reported that the SSRI sertraline enhanced the effect of a visual stimulation protocol designed to induce long-term potentiation (LTP) in the human primary visual cortex. Furthermore, SSRIs can enhance motor cortex excitability and plasticity (Loubinoux et al., 1999, 2002a,b, 2005; Gerdelat-Mas et al., 2005). For example, a single dose of citalopram induced acute increases in motor cortex plasticity measured with paired associative stimulation (PAS), a technique that utilizes transcranial magnetic stimulation (TMS) paired with electrical stimulation of a peripheral nerve to induce LTP like changes in cortical excitability (Bezchlibnyk-Butler et al., 2000). Fluoxetine may also enhance the effects of physiotherapy for stroke patients, possibly by increasing motor cortex plasticity (Chollet et al., 2011).

The primary aim of this double blind, placebo-controlled study was to assess whether a 3-week course of fluoxetine would enhance VPL of a motion direction discrimination (MDD) task (Ball and Sekuler, 1987) in visually normal adults. Our hypothesis was that fluoxetine would enhance the rate, amount and transfer of VPL by increasing visual cortex plasticity.

The study also involved a number of secondary aims. We assessed the effect of SSRIs on visual and motor cortex excitability by using TMS to measure phosphene and motor thresholds (Deblieck et al., 2008). Acute changes in excitability were measured 2 h after a single dose of citalopram, an SSRI with a rapid onset of action (Bezchlibnyk-Butler et al., 2000). Chronic changes in excitability were assessed during and after the course of fluoxetine. Also, acute and chronic SSRI effects on motor cortex plasticity were assessed using PAS. Finally, at the end of the study, we delivered an acute dose of the benzodiazepine triazolam to test the effect of increased GABA mediated inhibition on MDD task performance (both trained task performance and transfer of learning). Genotyping for BDNF was also conducted for a subset of our participants because BDNF may influence an individual’s response to interventions designed to modulate neuroplasticity (Cheeran et al., 2010).

## Materials and Methods

### Study Design

A double blind, placebo controlled experimental design was adopted. Participants were randomized to the active or placebo group by a computer-based random number generator after recruitment to the study. Investigators were masked to group allocations until data collection and processing were complete.

The study design is shown in **Table 1**. Participants completed 14 study visits over a period of 6 weeks. The first two visits were practice sessions in which participants were familiarized with the psychophysical MDD task and the use of TMS to measure visual and motor cortex excitability via phosphene and motor thresholding techniques. Session 3 involved baseline measurements and session 4 repeated these measures after an acute dose of citalopram (active group) or placebo (placebo group). Citalopram was chosen for the assessment of acute SSRI effects because its peak plasma concentration is reached after ∼2 h rather than fluoxetine which takes in excess of 6 h. The day after session 4 participants began their 19-day course of once-daily fluoxetine (active group) or placebo (placebo group) tablets. Fluoxetine was used for this section of the study because it is has a longer half-life than citalopram (fluoxetine = 1–3 days for an acute dose vs. 35 h for citalopram). This results in consistent plasma concentrations and therefore more stable pharmacodynamic effects. A 19-day course was provided because the downstream auto-regulatory effects of the SSRIs take around 2 weeks to occur (Stahl, 1998). Measures were repeated on the day of the 7th dose (session 5) and on the day of the 14th dose (session 6). A course of five daily VPL sessions (sessions 7–11) began on the day of the 15th dose and ended on the day of the 19th and final dose. Measures were made the day after the final dose (session 12) and a blood sample was taken for BDNF genotyping. Three days after the final fluoxetine dose all participants (both active and placebo groups) were given triazolam (time to peak plasma concentration ∼1 h, biological half-life = 2 h) and measures were repeated 2 h later (session 13). A final set of measures was collected 1 week after the triazolam dose (session 14).

**Table 1:** The study design.

	Week 1	Week 2	Week 3	Week 4	Week 5	Week 6
Monday		S3: Baseline MDD, TMS			S10: VPL	
Tuesday					S11: VPL End fluox/pla	
Wednesday					S12: Post VPL MDD, TMS	
Thursday	S1: Prac MDD, TMS	S4: Cital/pla MDD, TMS	S5: Wk 1 MDD, TMS	S6: Pre VPL MDD, TMS		S14: Washout MDD, TMS
Friday	S2: Prac MDD, TMS	Start fluox/pla		S7: VPL	S13: Triazolam MDD, TMS	
Saturday	–			S8: VPL		
Sunday	–			S9: VPL		

*Prac, practice session; MDD, motion direction discrimination psychometric function measurements; TMS, transcranial magnetic stimulation measurements (phosphene and motor thresholds); cital, citalopram; pla, placebo; fluox, fluoxetine, gray shading indicates daily doses of fluoxetine or placebo; VPL, visual perceptual learning training. The Profile of Mood States Short Form Questionnaire was administered at every study visit.*

### Participants

Twenty participants were recruited using posters and advertising in and around the University of Auckland. Inclusion criteria: male, right-handed [Edinburgh Handedness Questionnaire (Oldfield, 1971) score greater than zero], 18–40 years of age, normal or corrected-to-normal vision, no contraindications for TMS. Exclusion criteria: self-reported personal or family history of a mood disorder such as depression or bipolar disorder, smoker, diabetes, history of: drug, alcohol or nicotine addiction, use of medications or supplements known to alter mood; such as St John’s Wort, history of seizures, post-graduate students supervised by one of the researchers involved in the study. The Northern X Ethics Board of New Zealand approved the study and all study protocols compiled with the Declaration of Helsinki.

Right-handed males were recruited to reduce variability in the measurement of motor cortex excitability due to handedness or hormonal changes during the menstrual cycle. A psychiatrist (SSRI eligibility) and a neurologist (TMS eligibility) reviewed screening information prior to enrollment. Participants were paid $600 in vouchers and were asked to refrain from taking caffeine on test days and avoid the use of recreational drugs (except alcohol) during the study. A urine test was administered as part of session 12 to screen for recreational drug use. No participants failed the test. Regular telephone contact was maintained with participants to ensure that they did not experience any adverse drug effects and to encourage compliance with the study protocol.

### Drug Prescription

Active or placebo tablets (1 × 20 mg citalopram and 19 × 20 mg fluoxetine or 20 × methylcellulose) were dispensed in unmarked blister packs by an unmasked pharmacist. A 3-week course was chosen to limit the exposure of our participants to SSRIs. All participants were given 0.0625 mg of triazolam at the start of session 14. The citalopram and triazolam doses were taken 2 h before testing. Fluoxetine doses were taken at breakfast.

### Motion Direction Discrimination

**FIGURE 1:**
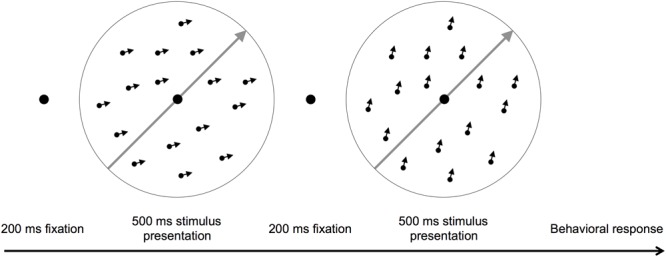
**A schematic of the motion direction discrimination task.** The gray arrows show the motion orientation and the small black arrows individual dot directions. Participants judged whether two consecutively presented dot fields moved in the same or different directions. The arrows are for illustration purposes only.

Participants fixed on a central point and judged whether the motion direction of two consecutively presented dot fields was the same or different (Ball and Sekuler, 1987; Liu, 1999). The stimulus contained 400 dark dots (0.1°) randomly distributed within a light gray 8° circular aperture (65 cm viewing distance). During each stimulus presentation, the dots moved coherently above or below a fixed motion orientation, which was either toward the top right hand corner (315° orientation) or the top left hand corner of the screen (225° orientation) at 10°/s. If the stimuli moved in different directions, one direction was always above the fixed motion orientation and one was below. Therefore, the motion stimulus had two direction components, a motion orientation and a motion direction (**Figure 1**). The motion orientation bisected the two motion directions shown within a trial. Task difficulty was manipulated by varying the angular difference between the two directions shown in a trial.

Psychometric functions were measured without feedback separately for each motion orientation during sessions 3–6 and 12–14. A psychometric function for one motion orientation consisted of 140 trials for each of five angular differences (12°, 10°, 8°, 6°, and 4°) that were presented in a random order (700 trials total). A Weibull function was fitted to the resulting data for each motion orientation to provide an estimate of the 75% correct threshold angular difference.

Participants were familiarized with the task in sessions 1 and 2 by performing 40 trials at an angular difference of 15° with visual feedback until they reached 90% accuracy for both motion orientations. Two psychometric function measurements were then completed with feedback, one for each motion orientation.

Motion direction discrimination training occurred on sessions 7–11 and involved a daily block of 700 trials with feedback presented at the 75% threshold measured on session 6 (the day before training). Only one motion orientation was trained. The motion orientation to be trained was randomly assigned to each participant prior to active or placebo group randomization.

### Mood

The Profile of Mood State – Short Form questionnaire (POMS-SF), and the Depression, Anxiety and Stress Scale (DASS-21) were administered at the initial screening and consent visit as part of the eligibility criteria assessment. The POMS-SF was completed at every session to test for any SSRI-induced changes in mood. The Total Mood Disturbance score (TMD = [Tension + Depression + Anger + Fatigue + Confusion] – Vigor) was used for analysis (McNair et al., 1971).

### Transcranial Magnetic Stimulation

Transcranial Magnetic Stimulation measures of visual and motor cortex excitability were made on sessions 1–6 and 12–14 using a MagStim 200 stimulator and a flat, 70 mm figure of eight coil. PAS was delivered using a MagStim Rapid 2 stimulator equipped with a 70 mm double air film coil and a Grass S48 direct current stimulator with a constant current unit (CCU5) was used for median nerve stimulation.

### Phosphene Thresholds

Participants wore lightproof goggles and were instructed to keep their eyes open, while gazing straight ahead. Single-pulse TMS was delivered with the coil handle oriented upward. The coil center was positioned 2 cm above the inion, and the coil position was altered in 1 cm steps in a grid pattern until the optimal position for eliciting a phosphene was determined. Stimulation began at 30% maximum stimulator output (MSO) and was increased until a phosphene was observed reliably. If 100% MSO was reached on two attempts without phosphene perception the procedure was terminated. Phosphenes were verified by ensuring that they occurred in the hemifield contralateral to the stimulated hemisphere. The phosphene threshold was defined as the minimum intensity that produced a phosphene on four out of eight consecutive TMS pulses.

In addition to a standard phosphene threshold, a new measure of perceived phosphene intensity was used to quantify any SSRI-induced changes in the suprathreshold phosphene stimulus-response function. A stimulus intensity range was calibrated using the immediately preceding phosphene threshold measurement. Ranges were 40–80, 50–90, or 60–100% MSO with pulses delivered at 10% MSO intervals within the range. The range with the lower bound closest to (but exceeding) the phosphene threshold was chosen on a session-by-session basis. First, a pulse with the strongest strength in the range was delivered to the phosphene hotspot to generate a reference phosphene. Participants rated the strength of all subsequent phosphenes relative to the reference using a visual analogue scale (VAS). Each of the five pulse strengths was repeated five times in a random order. Perceived intensity was calculated by measuring the distance between the start of a VAS line and the bisection drawn by the participant in mm. Data from each session were fit with a logistic function to provide a 50% of maximum intensity threshold. Thresholds exceeding 100% MSO were set to 100% MSO.

### Resting Motor Threshold

Electromyography (EMG) was recorded in a standard manner by using electrodes placed on the abductor pollicis brevis (APB) muscle and the first dorsal interosseous (FDI) muscle, of the relaxed right hand. Rest motor threshold (RMT) was determined for the FDI muscle to the nearest 1% of stimulator output as the lowest stimulus intensity that produced an MEP > 50 μV in at least four of eight trials (Rossini et al., 1994).

### Paired Associative Stimulation

Motor cortex excitability was assessed pre- and post-PAS by measuring stimulus response curves for both the APB and FDI muscles. Single pulse TMS was delivered in pseudo randomized blocks of 10 trials at stimulation intensities of 40, 50, 60, 70, and 80% MSO. For each trial, peak to peak MEP amplitude was determined within a 10–45 ms window following TMS. Pre-TMS root mean square EMG activity was recorded for 100 ms prior to each TMS pulse to ensure that the muscles were at rest. The MEP amplitudes within a block were ranked and the mean MEP amplitude for the central six MEPs was calculated. A linear function was then fit to the means of the five blocks within a session and the slope of the function was used as a measure of excitability. The ratio of pre to post PAS slopes (post/pre) was used for analysis.

The PAS protocol involved direct current stimulation of the right median nerve combined with motor cortex TMS. Median nerve stimulation had an intensity of three times the participant’s sensory detection threshold and was delivered in bursts of 10 pulses at 30 Hz. Bursts of median nerve stimulation were paired with TMS of the APB hotspot once every 1.1 s. TMS was delivered at 100% of RMT. The PAS protocol delivered 180 pairs of stimuli (Stefan et al., 2002) and lasted 200 s.

### BDNF Polymorphisms

Participants were asked to provide a blood sample at the end of session 12. The sample was processed to identify single-nucleotide polymorphisms (SNP) for BDNF. Participants could opt out of the blood sample and still remain eligible for the study. Genotyping was undertaken using the Agena MassArray iPLEX assay (Agena Bioscience, San Diego, CA, USA). Analysis using the Bruker Mass Spectrometer was then conducted using parameters optimized for iPLEX chemistry allowing allele specific single base extensions to be resolved. Visual quality checks were made of the generated peaks in addition to the non-template control prior to report generation using Typer 4 analysis software (Agena Bioscience).

## Results

Twenty participants completed the study, 10 active (mean age 24.5 years, ±6.1) and 10 placebo (24.5, ±5.5 years). Three eligible participants withdrew from the study prior to randomization due to the time commitment required. Three participants (two active and one placebo) did not attend session 4 due to mild illnesses such as colds. The drug dose was still taken. BDNF polymorphisms were available for 14 participants (seven per group). Both groups included participants with each possible BDNF polymorphism (Placebo; 3 val66, 1 met66, and 3 val66met, Active; 4 val66, 1 met66, and 2 val66met). ANOVA analyses were Huynh-Feldt corrected for sphericity where necessary.

### Visual Perceptual Learning

#### Fluoxetine and Learning

**FIGURE 2:**
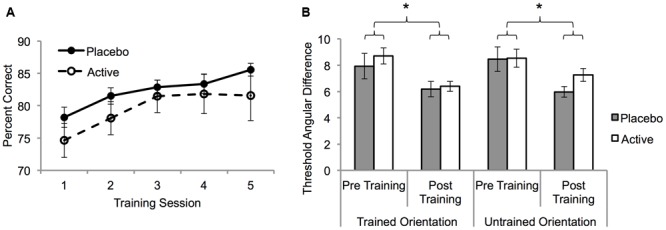
**The effect of fluoxetine on visual perceptual learning (VPL). (A)** Group mean trained task percent correct for the placebo and active groups as a function of training session (S7–S11: **Table 1**). Task difficulty was fixed at the pre training 75% correct threshold. **(B)** 75% correct angular difference thresholds for the trained and untrained motion orientations from psychometric function measurements made pre (S6) and post (S12) training (**Table 1**). Error bars show SEM. Asterisks denote statistically significant main effects of training (*p* < 0.05) see main text for details.

A mixed ANOVA with factors of treatment group (active vs. placebo) and training session (five levels; sessions 7–11), was conducted on the percent correct scores. Accuracy improved across sessions for both groups (*F*_3,47_ = 11.4, *p* < 0.001; **Figure 2A**). However, there was no interaction between session and treatment group (*F*_3,47_ = 0.51, *p* = 0.7) indicating that the rate and magnitude of learning did not differ between the two groups. To confirm this, linear functions were fit to each participant’s learning curve and the slopes were compared between the active and placebo groups. The mean slope for the active group (1.7, ±1.2) did not differ from the placebo group (1.8, ±2.4), *t*_18_ = 0.1, *p* = 0.9. In addition, exponential functions were fit to the error rate curves for each participant and the model parameters were compared between the active and placebo groups. No significant differences were found between the two groups (all *t* > 1.1, all *p* > 0.1). A separate ANOVA with an additional between subjects factor of BDNF polymorphism (*n* = 14) revealed no main effects or interactions involving BDNF polymorphism (all *p* > 0.05).

To determine the specificity of learning for the trained motion orientation, psychometric functions before (session 6) and after (session 12) training were compared. A mixed ANOVA with factors of treatment group, session (pre-training, post-training) and motion orientation (trained, untrained) was conducted on the angular threshold data. Learning significantly reduced thresholds (*F*_1,18_ = 22.6, *p* < 0.001) but the extent of this learning was not influenced by group or motion orientation (all interactions *p* > 0.05; **Figure 2B**). These results demonstrate a full transfer of learning from the trained motion orientation to the untrained motion orientation that did not differ significantly between the two groups.

#### Acute Effect of Citalopram on Motion Direction Discrimination

Angular thresholds derived from psychometric function measurements were compared between the baseline session (Session 3) and the acute citalopram session (Session 4). The data were collapsed across motion orientation as training had not yet taken place. A repeated measures ANOVA with factors of session (baseline and acute citalopram) and group (placebo vs. active) revealed that citalopram had no acute effect on task performance. There was no significant main effect of session (*F*_1,15_ = 1.3, *p* = 0.3), between-subjects effects of group (*F*_1,15_ = 195.5, *p* = 0.3) or interaction (*F*_1,15_ = 3.8, *p* = 0.07).

#### Triazolam and Post-training Motion Direction Discrimination

Angular thresholds were compared the day before (session 12), 2 h after (session 13), and the 1-week after (session 14) triazolam administration. Two participants were excluded from this secondary analysis (one from each group) as they did not demonstrate learning over the 5-day training period and therefore the effect of triazolam on improved task performance could not be measured. These two participants showed no improvement in task performance between the first and last training session and linear functions fitted to their training data were negative.

**FIGURE 3:**
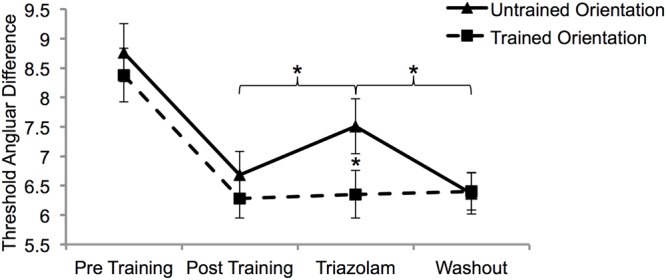
**The effect of triazolam on trained and untrained task performance after learning was complete.** There was complete transfer of learning from pre (S6) to post (S12) training. Only learning transfer was affected by triazolam (S13). Thresholds had returned to post training levels 1 week after triazolam (washout; S14). Error bars show SEM. Asterisks depict statistically significant effects of triazolam (*p* < 0.05).

A mixed ANOVA with factors of session (post-training, triazolam, and washout), training orientation (untrained and trained) and group (active and placebo) was conducted on the angular difference thresholds. Only the interaction between training orientation and session was significant (*F*_2,32_ = 3.4, *p* = 0.04). Paired samples *t*-tests conducted on the angular difference thresholds collapsed across group demonstrated that triazolam impaired MDD for the untrained orientation only (**Figure 3**).

#### Cortical Excitability

**FIGURE 4:**
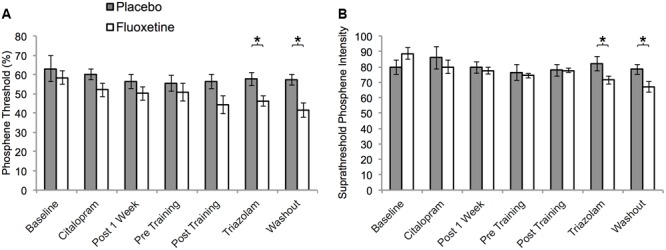
**Visual cortex excitability as a function of session for participants who perceived phosphenes within the placebo (*n* = 8) and active (*n* = 5) groups. (A)** Phosphene thresholds. **(B)** Suprathreshold phosphene intensities corresponding to 50% of maximum intensity. Units are % maximum stimulator output (MSO). Sessions: baseline = S3, citalopram = S4, Post 1 week = S5, Pre Training = S6, Post Training = S12, Triazolam = S13, Washout = S14. Asterisks indicate statistically significant differences between the active and placebo groups (*p* < 0.05).

Thirteen participants (eight placebo, five active) perceived phosphenes and were included in the phosphene threshold analyses. One participant from the placebo group had a missing data point (S4) that was imputed using the last observation carried forward method. For standard phosphene thresholds, a mixed ANOVA with factors of session (seven sessions: 3–6 and 12–14) and group (placebo and active) revealed a significant main effect of session (*F*_4,43_ = 5.8, *p* = 0.001) indicating a systematic reduction in phosphene threshold over time (**Figure 4A**). There was no session by group interaction (*F*_4,43_ = 2.0, *p* = 0.11). A close inspection of the data (**Figure 4**) suggested a trend for a larger decrease in threshold for the active group across sessions. The active group had significantly lower thresholds than the placebo group on session 13 (*t*_11_ = 2.7, *p* = 0.02) and session 14 (*t*_11_ = 3.3, *p* = 0.01).

A similar pattern of results was evident for the suprathreshold phosphene intensity rating measurements (**Figure 4B**). Thresholds significantly decreased over consecutive sessions (*F*_6,66_ = 3.1, *p* = 0.008) indicating an increase in cortical excitability. This effect differed significantly between the two groups (significant interaction between treatment group and session, *F*_6,66_ = 2.5, *p* = 0.03). The placebo group did not exhibit a significant change over consecutive sessions (*F*_6,42_ = 0.3, *p* = 0.9), whereas the active group did (*F*_6,24_ = 7.1, *p* < 0.001). The groups differed significantly at sessions 13 (*t*_11_ = 2.1, *p* = 0.04) and 14 (*t*_11_ = 2.4, *p* = 0.04).

**FIGURE 5:**
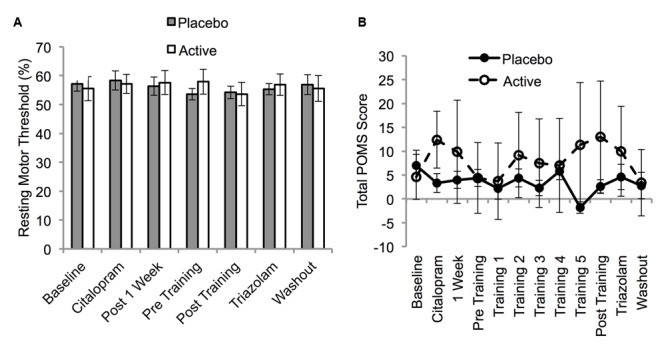
**Resting motor threshold **(A)** and Profile of Mood States Short Form (POMS) Total Score **(B)** as a function of session for the placebo and active groups.** Resting motor thresholds are expressed in units of % MSO and were assessed on sessions 3–6 and 12–14 (**Table 1**). POMS scores were collected on every session. Error bars show SEM.

Resting motor thresholds were stable across all sessions (*F*_6,108_ = 0.72, *p* = 0.6) and did not differ between the two groups for any session (*p* > 0.05; **Figure 5A**).

#### Mood

Six participants in the placebo group and four participants in the active group had one missing POMS score due to an absence or incorrect completion of the questionnaire. These missing data points were imputed using the last observation carried forward when possible. If the S3 data point was missing, the S4 data point was carried backward. The POMS-SF TMD score did not vary significantly across sessions (*F*_4,73_ = 0.71, *p* = 0.6) and there was no session by group interaction (*F*_4,73_ = 1.36, *p* = 0.2; **Figure 5B**). This was also the case when the analyses were repeated without imputation for missing values. Therefore, consistent with previous work, (Gelfin et al., 1998) [but see (Serretti et al., 2010)] fluoxetine did not alter mood in our healthy participants.

#### Paired Associative Stimulation

**FIGURE 6:**
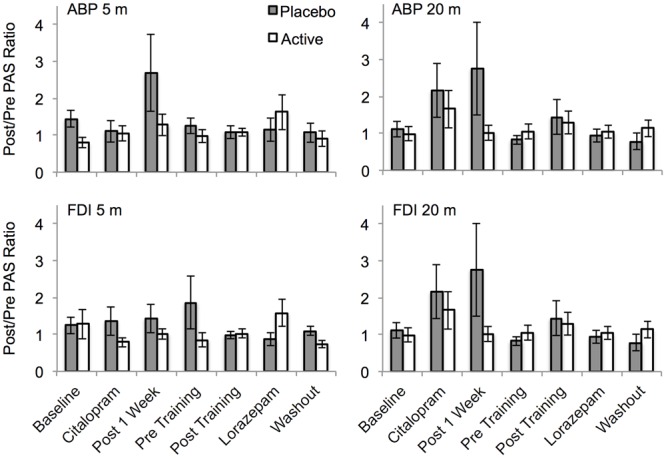
**The effect of PAS on motor cortex excitability across sessions for the placebo and drug groups.** PAS data are shown as linear slope of the stimulus response curve post/pre ratios. Ratios of 1 indicate no change, >1 indicates facilitation, <1 indicates suppression. APB, abductor pollicis brevis muscle; FDI, first dorsal interosseous muscle; 5 m, 5 min post PAS; 20 m, 20 min post PAS. Error bars show SEM.

There was considerable variability in the response to PAS (**Figure 6**). Significant PAS induced facilitation was not observed at baseline for either muscle (APB or FDI) or post PAS time point (5 and 20 min post PAS), all *t* < 1.2, all *p* > 0.25. The placebo and active groups did not differ significantly across sessions for either post PAS time point for either muscle (all *F* < 1.8, all *p* > 0.1). There were no interactions between group and session (all *F* < 0.2, all *p* > 0.2). A sub-analysis including only participants for whom BDNF polymorphism data were available revealed no interaction between BDNF polymorphism and group (all *F* < 0.6, all *p* > 0.4). A separate analysis of the pre and post citalopram data (session 3 vs. session 4) revealed no significant interaction between session and group for either the 5 min post PAS (*F*_1,15_ = 0.2, *p* = 0.7) or the 20 min post PAS data (*F*_1,15_ = 1.6, *p* = 0.2).

## Discussion

Our primary hypothesis was that fluoxetine would increase the rate and magnitude of VPL compared to placebo in observers with normal vision. This hypothesis was not supported. The active and placebo groups did not differ significantly in any of the metrics of VPL that we investigated. However, increased GABA-mediated inhibition due to an acute dose of triazolam was found to selectively impair improved MDD that was due to learning transfer. This suggests that training and transfer effects induced by VPL rely on dissociable neural mechanisms.

Studies designed to pharmacologically manipulate learning are faced with a large parameter space in terms of drug dosing. Our choice of 20 mg per day for 3 weeks was driven by a desire to give the minimum clinically effective dose for a period of time that does not typically affect mood. However, it is possible that larger doses or longer courses of fluoxetine would enhance learning. Prior studies on rats have used high doses of fluoxetine to induce plasticity (Maya Vetencourt et al., 2008). It is also possible that SSRIs may have a greater influence on visual cortex plasticity in patients with visual disorders such as amblyopia. A greater propensity for perceptual learning has been reported in such patients (Huang et al., 2008). Finally, we may not have had sufficient statistical power to detect a difference in learning between the two treatment groups. However, our data do not show any trends that are indicative of an effect of fluoxetine on learning suggesting that increasing statistical power may not have changed our results.

We observed a complete transfer of learning to the untrained motion orientation after 5 days of training. This was unexpected based on previous studies using the same task (Ball and Sekuler, 1987). Two factors are likely to have contributed to this transfer. Firstly, participants were tested extensively on both motion orientations prior to training over the course of five psychometric function measurements per orientation. Each measurement consisted of 700 trials. Familiarization to both motion orientations also occurred in the initial practice sessions. This procedure ensured a stable pre-training baseline measure for both motion orientations. Pre-training or double training has previously been found to prime transfer to untrained stimulus features (Xiao et al., 2008) including an untrained motion direction (Zhang and Yang, 2014). Our psychometric function measurements may have acted to prime transfer to the untrained motion orientation. Second, we trained participants at a fixed angular difference that corresponded to their 75% correct threshold pre training and allowed accuracy to improve. This meant that the task became easier and required less precision. Training on easier tasks (Liu, 1999) results in greater learning transfer and transfer is greater to tasks that do not require high precision judgments (Jeter et al., 2009). In addition, unlike some previous studies, (Liu and Vaina, 1998; Rokem and Silver, 2010) the corners of the monitor screen were visible to our participants during stimulus presentation. Although the stimulus was presented centrally and was 13.5° from the corners of the screen, it is possible that the corners provided spatial cues that aided learning transfer.

The complete transfer of learning between the trained and untrained motion orientations enabled observation of an unexpected effect of the benzodiazepine triazolam on MDD task performance. Triazolam significantly impaired task performance for the untrained motion direction (learning transfer) but not the trained motion direction. To our knowledge, this is the first pharmacological evidence for differential neural mechanisms underlying learning and transfer in a VPL paradigm. The differential effect of triazolam on learning and transfer is consistent with current models of perceptual learning that postulate a two-stage learning process for the trained task (changes at both a sensory and decision making stage) and a single stage learning process for transfer (changes at a decision making stage only) (Watanabe and Sasaki, 2015). Triazolam causes hyperpolarization of cells by allosteric modulation of the GABA–A binding site and therefore inhibits neural activity. Triazolam reduces the temporal sensitivity of vision (Maddock et al., 1993) and similar benzodiazepines such as lorazepam may impair visual processing and attention mechanisms (Giersch et al., 1995; Giersch and Herzog, 2004; Michael et al., 2007). Our data indicate that the neural mechanisms underlying VPL of the trained stimulus are robust to the effects of triazolam whereas those underlying learning transfer are not. This may reflect a stronger effect of triazolam on higher-level areas that underpin learning transfer compared to early sensory processing areas that support trained task performance.

Selective serotonin reuptake inhibitors have been found to increase cortical excitability (Loubinoux et al., 1999, 2002a, 2005; Gerdelat-Mas et al., 2005). We did not find significant acute effects of citalopram on visual cortex excitability, however, chronic administration of fluoxetine did appear to increase excitability, particularly for the suprathreshold phosphene intensity measure. However, the lack of a session by group interaction effect for the phosphene threshold measure and the fact that not all participants saw phosphenes preclude strong conclusions being drawn. We found no evidence for SSRI induced changes in resting motor threshold. This is inconsistent with previous reports. For example, a single dose of citalopram has been reported to transiently reduce motor thresholds (Robol et al., 2004). Previous studies have compared pre and post citalopram measures that were made on the same day, whereas, we compared the post citalopram measurements to measurements made 2 days prior. The added variability within our design may have masked any small effect of citalopram on motor cortex excitability.

We did not observe statistically significant PAS induced facilitation of cortical excitability for the group in any session of this study, nor did we observe any effect of SSRI administration. There are considerable individual differences in the response to PAS (Stefan et al., 2004; Cheeran et al., 2008; Cirillo et al., 2009, 2012; Huang et al., 2009; Missitzi et al., 2011; Witte et al., 2012) and it is possible that our relatively small sample size did not provide sufficient power to detect PAS effects. Alternatively, the protocol utilized may not have been optimal to induce LTP-like facilitation of cortical excitability.

In summary, we found no evidence for an enhancement of motion VPL with fluoxetine. However, triazolam administration impaired task performance for an untrained motion orientation that exhibited learning transfer but not for the trained motion orientation. This provides support for the theory that learning and transfer of learning rely on dissociable neural mechanisms.

## Author Contributions

Experiment design: AL, JB, WB, RK, BR, CS, and BT. Data collection: AL, MF, and LG. Data analysis: AL and BT. Manuscript preparation: AL and BT. Manuscript Editing: JB, WB, RK, BR, CS, and MF. Project supervision: BT.

## Conflict of Interest Statement

The authors declare that the research was conducted in the absence of any commercial or financial relationships that could be construed as a potential conflict of interest.
